# Aquarium Nitrification Revisited: Thaumarchaeota Are the Dominant Ammonia Oxidizers in Freshwater Aquarium Biofilters

**DOI:** 10.1371/journal.pone.0023281

**Published:** 2011-08-16

**Authors:** Laura A. Sauder, Katja Engel, Jennifer C. Stearns, Andre P. Masella, Richard Pawliszyn, Josh D. Neufeld

**Affiliations:** Department of Biology, University of Waterloo, Waterloo, Ontario, Canada; Argonne National Laboratory, United States of America

## Abstract

Ammonia-oxidizing archaea (AOA) outnumber ammonia-oxidizing bacteria (AOB) in many terrestrial and aquatic environments. Although nitrification is the primary function of aquarium biofilters, very few studies have investigated the microorganisms responsible for this process in aquaria. This study used quantitative real-time PCR (qPCR) to quantify the ammonia monooxygenase (*amoA*) and 16S rRNA genes of Bacteria and Thaumarchaeota in freshwater aquarium biofilters, in addition to assessing the diversity of AOA *amoA* genes by denaturing gradient gel electrophoresis (DGGE) and clone libraries. AOA were numerically dominant in 23 of 27 freshwater biofilters, and in 12 of these biofilters AOA contributed all detectable *amoA* genes. Eight saltwater aquaria and two commercial aquarium nitrifier supplements were included for comparison. Both thaumarchaeal and bacterial *amoA* genes were detected in all saltwater samples, with AOA genes outnumbering AOB genes in five of eight biofilters. Bacterial *amoA* genes were abundant in both supplements, but thaumarchaeal *amoA* and 16S rRNA genes could not be detected. For freshwater aquaria, the proportion of *amoA* genes from AOA relative to AOB was inversely correlated with ammonium concentration. DGGE of AOA *amoA* genes revealed variable diversity across samples, with nonmetric multidimensional scaling (NMDS) indicating separation of freshwater and saltwater fingerprints. Composite clone libraries of AOA *amoA* genes revealed distinct freshwater and saltwater clusters, as well as mixed clusters containing both freshwater and saltwater *amo*A gene sequences. These results reveal insight into commonplace residential biofilters and suggest that aquarium biofilters may represent valuable biofilm microcosms for future studies of AOA ecology.

## Introduction

Ammonia is a toxic metabolic waste product excreted by fish and other aquatic organisms. Ammonia toxicity can threaten aquatic ecosystem health and is a particular concern for relatively closed ecosystems, such as aquaculture operations and home aquaria, in which ammonia can quickly accumulate to lethal concentrations in the absence of active nitrification. The un-ionized form of ammonia (NH_3_) is particularly toxic to fish; stress, disease, and death may be associated with concentrations that exceed 0.1 mg L^−1^ in aquarium and aquaculture systems [1], [2]. In order to convert ammonia to nitrate, aquarium biofilters are designed to promote the growth and activity of nitrifying populations due to the high surface area of filter support material (e.g. sponge, ceramic or polymer) and rapid flow rates of aerated water. Despite their importance to fish health and identical function within many industrial biofilters, including aquaculture and wastewater treatment, little is known of the microorganisms catalyzing nitrification in association with aquarium biofilter support material.

Before the discovery of ammonia-oxidizing archaea (AOA), belonging to the newly proposed phylum Thaumarchaeota [3], [4], molecular approaches were used to investigate ammonia-oxidizing bacteria (AOB) and nitrite-oxidizing bacteria in freshwater and marine aquaria [5], [6]. In particular, Hovanec and DeLong used oligonucleotide probes to target bacterial nitrifiers in freshwater and saltwater aquarium biofilter DNA extracts. Although *Nitrosomonas*-like bacteria from the *Betaproteobacteria* were associated with the saltwater aquaria in their study, they did not detect these bacteria in most of the freshwater aquarium biofilter extracts tested. They concluded that “the bacterial species responsible for nitrification in simple freshwater systems remain unknown” [5]. Subsequent studies determined that *Nitrosomonas* spp. could indeed be enriched from freshwater aquarium biofilters [7], suggesting their potential involvement in ammonia oxidation under *in situ* conditions. Since the discovery of AOA, two studies have investigated the presence and diversity of both AOA and AOB in marine biofiltration systems [8], [9]. Foesel and colleagues found that *Nitrosomonas*-like AOB were numerically dominant in a marine aquaculture biofilm. Urakawa and colleagues identified the presence of *amoA* genes from AOB and AOA in selected marine aquarium biofilters from a public aquarium in Japan (i.e. sunfish tank, cold water tank, and a coastal fish tank), and suggested that the diversity of AOA and AOB was decreased in low temperature marine aquaria. *Candidatus* Nitrosopumilus maritimus SCM1, the first AOA representative isolated in pure culture, was obtained from saltwater aquarium gravel [10]. Despite these initial studies, no research has yet investigated the abundance of AOB and AOA in freshwater aquarium biofilters.

Based on the ubiquity and high abundance of AOA in natural environments, the inability of Hovanec and DeLong (1996) to detect AOB in freshwater aquaria, and the isolation of the first ammonia-oxidizing archaeon from aquarium substrate [10], we hypothesized that AOA dominate freshwater aquarium biofilters and play an important role in aquarium nitrification. In addition to determining the abundances of AOA and AOB in aquaria, the objectives of this study were to assess the diversity of AOA *amoA* genes in aquaria, and to determine how these genes clustered with sequences derived from environmental sources and cultured AOA representatives. The results of this study revealed that, based on *amoA* gene abundances, AOA were the dominant putative ammonia oxidizers in the majority of freshwater and saltwater aquaria. These results provide first evidence for the important role of AOA in freshwater aquarium filtration and suggest possible niche adaptation of AOA to conditions associated with freshwater aquarium biofilters.

## Results

### Aquarium samples

Twenty-seven freshwater and eight saltwater aquarium filter samples were collected from retail and residential locations in three cities (Table S1). All biofilters sampled in this study were derived from standalone aquaria in homes (or offices), or from display tanks in retail outlets, reflecting conditions common to most residential or retail aquaria, respectively. In addition to aquarium biofilters, we included two aquarium supplements in the analysis. The aquaria selected for sampling ranged in pH from 7.6 to 9.2 and varied in their fish and live plant composition. The aquaria contained a variety of fish including mixed tropical, goldfish, South American cichlids and African cichlids. Three aquaria had received antibiotic treatment in the previous six months (SW4, SW5, FW8) and several were known to have received doses of bacterial filter supplement when first established (e.g. FW12, FW13, FW19, FW25). Ammonium concentrations of aquaria ranged from below detection to approximately 0.5 mg L^−1^, with the majority of aquaria below 100 µg L^−1^. In 28 of the 32 aquaria studied, nitrite (NO_2_^−^) was below detection. As expected, significant positive correlations were observed between ammonium and nitrite concentrations (r = 0.48, p<0.05; Table S2) and nitrite and nitrate concentrations (r = 0.52, p<0.05; Table S2). Aquaria ranged in size from 5 gallons to greater than 400 gallons (for large retail show tanks) and approximate fish numbers ranged from zero (in a plant tank; FW3) up to 300 (FW11). Although not a perfect measure of fish biomass, the approximate number of fish per gallon was positively correlated with ammonium concentration (r = 0.60, p<0.001; Table S2). Water hardness of freshwater aquaria was as low as 25 ppm (in FW12, a breeding tank using softened water), however, the water hardness of 25 of 27 aquaria was >150 ppm. Hardness of saltwater samples could not be determined using the kit utilized. Alkalinity (i.e. carbonate alkalinity/hardness) ranged from below detection (e.g. FW24, FW17) to 300 ppm (e.g. SW1, FW3). Neither hardness nor alkalinity correlated significantly with any other water chemistry parameters (Tables S2 and S3).

### AOA and AOB abundances

**Figure 1:**
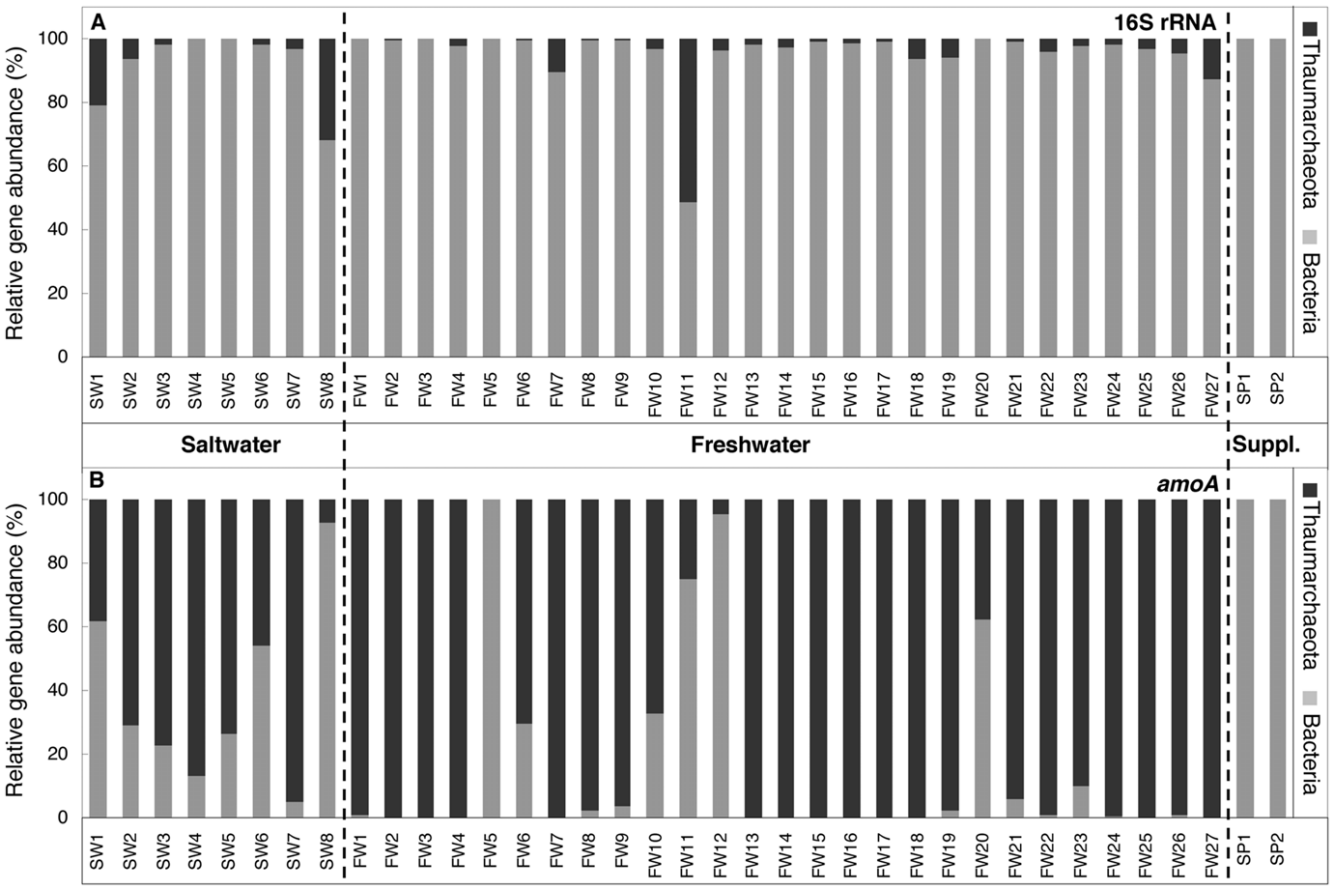
Relative gene abundances of Bacteria and Thaumarchaetoa in aquaria. Relative 16S rRNA (A) and *amoA* (B) gene abundances for bacteria and thaumarchaea in saltwater (SW1–SW8) and freshwater (FW1–F27) aquaria and in aquarium supplements (SP1, SP2). These data were calculated from duplicate qPCR amplifications.

Real-time PCR results demonstrated that thaumarchaeal *amoA* genes were dominant in 23 of the 27 sampled freshwater filters (Fig. 1; Table S1). For 12 of the freshwater biofilters, thaumarchaeal *amoA* genes represented the entire detected *amoA* gene signal, including FW13, FW15, FW16, and FW25, aquaria that received bacterial aquarium supplements when first established. For saltwater aquaria, *amoA* genes from both AOA and AOB were detected in all samples, with AOA dominating five of eight samples. For both commercially available aquarium supplements, AOB *amoA* genes were abundant, and both AOA *amoA* and 16S rRNA genes were below detection limits.

Both bacterial and thaumarchaeal 16S rRNA genes were detected in all aquarium DNA extracts; however, for the majority of samples, bacterial 16S rRNA genes greatly outnumbered thaumarchaeal 16S rRNA genes (Fig. 1; Table S1). In one aquarium biofilter (FW11), thaumarchaeal and bacterial 16S rRNA gene copy numbers were approximately equal. For all aquaria, bacterial *amoA* gene copy numbers were at least three orders of magnitude less than bacterial 16S rRNA genes (Table S1). In some aquaria (e.g. FW2, FW27, SW4), thaumarchaeal *amoA* and 16S rRNA gene copy numbers were approximately equal. In other cases (e.g. FW11, FW12, SW8), thaumarchaeal *amoA* gene copies were orders of magnitude less abundant than thaumarchaeal 16S rRNA genes.

**Figure 2:**
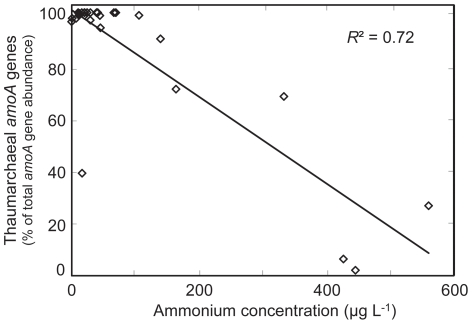
Freshwater aquaria ammonium concentrations and relative thaumarchaeal *amoA* gene abundances. AOA *amoA* gene copies are expressed as a percentage of the total *amoA* gene copies (per ng DNA). The coefficient of determination (*R^2^*) for the linear regression is 0.7249. The associated Pearson correlation coefficient (r) is −0.8518, with an associated p-value of <0.001. See Table S1 for all sample data.

We also examined aspects of water chemistry (Table S1) to assess correlations that might provide an explanation for differential *amoA* gene abundances. Regression analyses focused on freshwater aquarium samples because these were the primary focus of this study, and because too few saltwater samples were available to yield statistically significant correlations (data not shown). Correlations were also calculated that included both fresh and saltwater samples, which yielded similar results to freshwater correlations (Tables S2 and S3). For freshwater aquaria, we observed a significant negative correlation between ammonium concentrations and the proportion of *amoA* genes belonging to AOA rather than AOB (r = −0.85, p<0.001, *R* ^2^ = 0.72; Fig. 2 and Table S3). Low ammonia concentrations were typically associated with high AOA *amoA* gene abundances, although one sample (FW20) had high proportions of AOB *amoA* genes, despite an ammonia concentration of less than 20 µg L^−1^. In all cases, higher concentrations of ammonium were associated with higher relative abundances of AOB *amoA* genes. No other factors related to water chemistry or aquarium setup (e.g. pH, hardness, alkalinity) yielded significant correlations with *amoA* gene abundances (Table S2). Ammonia concentrations in aquarium FW27 fluctuated very little, both on daily and monthly scales (Fig. S1A). The high proportion (>85%) of AOA in this filter was also consistent over a two-year sampling period (Fig. S1B). Together, these results suggest temporal consistency in individual freshwater aquarium filter environmental conditions and AOA communities.

### AOA gene diversity

**Figure 3:**
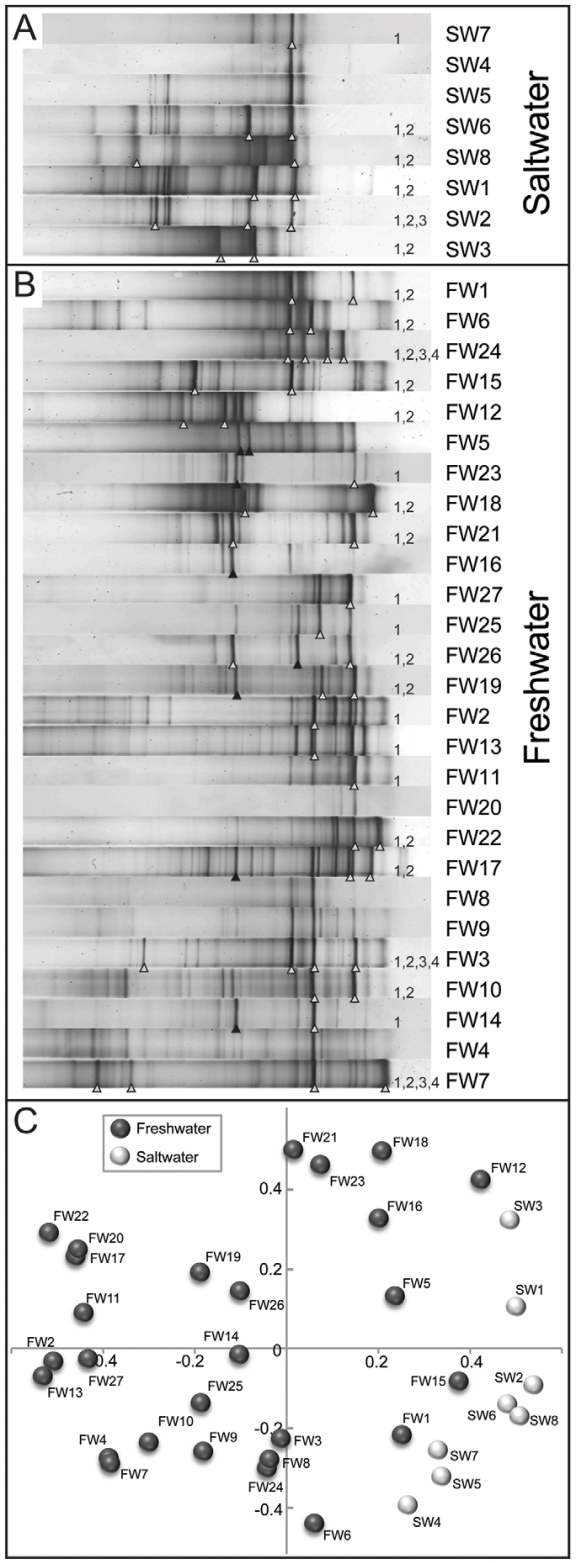
Denaturing gradient gel electrophoresis of thaumarchaeal *amoA* gene amplicons. Saltwater (A) and freshwater (B) fingerprints have been normalized and aligned. Bands chosen for sequencing are indicated with triangles: white triangles correspond to bands appearing in Figure 4. Black triangles represent failed sequencing reactions. Clustering of freshwater and saltwater fingerprints (C) is based on nonmetric multidimensional scaling (NMDS) using Pearson correlations of background-subtracted densitometric curves.

In order to assess the sample-to-sample variability of thaumarchaeal populations possessing *amoA* genes, all biofilter DNA extracts were subjected to DGGE analysis. Based on the physical separation of PCR products by sequence heterogeneity and G+C content [11], the patterns generated by *amoA* gene amplicons varied between samples from very simple with few bands (e.g. FW8, FW20, SW4) to relatively complex with many bands (e.g. FW10, FW15, SW1; Fig. 3). DGGE patterns revealed shared bands between aquarium biofilters from the same location (e.g. FW8 and FW9, FW25 and FW26, and SW1 and SW2), indicating that location-specific factors likely influenced the specific composition of AOA. Despite these location-specific similarities, distinct clustering of fingerprints was not observed based on location, and many bands were shared between multiple samples from various locations (Fig. 3A and B, Table S1). Based on visual inspection of aligned fingerprints, a possible shift in G+C content was observed between salt- and freshwater fingerprints, with saltwater bands typically melting at lower denaturant concentrations than the majority of freshwater sequences (Fig. 3A and B). This apparent difference in G+C content was supported by sequences from both clone libraries and sequenced DGGE bands. Freshwater *amo*A gene sequences from clone libraries had an average G+C content of 45.4%, versus 43.8% in saltwater clones. Sequences of DGGE bands showed a similar pattern, with average G+C contents of 45.5% and 44.0% for freshwater and saltwater bands, respectively. Although relatively small (∼1.5%), this difference in average G+C content between freshwater and saltwater sequences was statistically significant (p<0.0001) for both clones and DGGE sequences, as determined by unpaired *t* tests. Nonmetric multidimensional scaling (NMDS) using densitometric curves generated from DGGE fingerprints revealed that freshwater and saltwater fingerprints were largely separated in two-dimensional space. However, some freshwater samples (e.g. FW1, FW12, FW15) were intermediate to freshwater and saltwater clusters (Fig. 3C).

**Figure 4:**
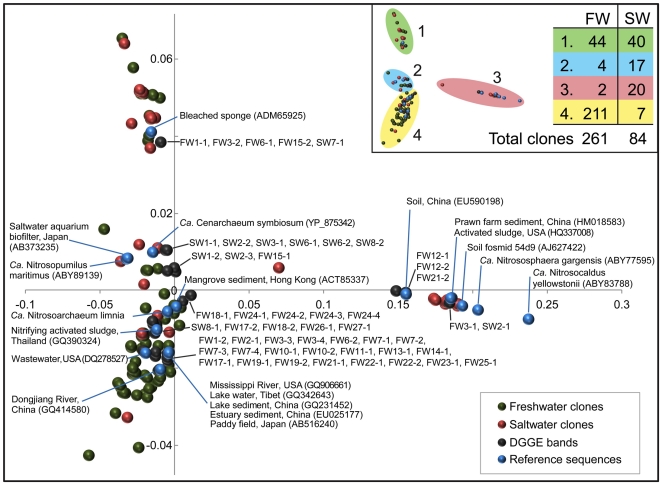
NMDS ordination of translated thaumarchaeal *amoA* gene sequences. Sequences were obtained from clone libraries and DGGE bands derived from freshwater and saltwater aquarium filter samples. The inset panel provides a summary of the number of freshwater and saltwater clone library sequences contained within each cluster. Sequences obtained from DGGE bands correspond to the white triangles in Figure 3, and are labelled with sample and band numbers. Selected Genbank sequences from uncultivated clones and reference strains are included for comparison. The sampling environment and Genbank accession numbers for all reference sequences are included within parentheses.

In addition to DGGE, we generated clone libraries of 261 and 84 sequences for freshwater and saltwater composite thaumarchaeal *amoA* gene PCR amplicons, respectively. Multidimensional scaling of translated and aligned *amoA* gene sequences (from DGGE bands, clone libraries, and reference sequences) revealed four distinct clusters, each of which contained both clones and DGGE band sequences (Fig. 4). The first cluster (cluster 1; Fig. 4) contained saltwater and freshwater clone library sequences in approximately equal proportions, as well as both saltwater and freshwater DGGE band sequences. The freshwater DGGE band sequences that fell into this cluster were generated from samples which had fingerprints that fell near the periphery of the freshwater cluster, close to saltwater sequences (Fig. 3C).

Both the second and third cluster (clusters 2 and 3; Fig. 4) were dominated by saltwater clone sequences. Cluster 2 was a distinctly saline cluster that contained *amoA* gene sequences from AOA originating from saline environments, including *Ca.* N. maritimus and *Candidatus* Cenarchaeum symbiosum A [YP_875342; 12], as well as an environmental clone obtained from a saltwater aquarium biofilter [AB373235; 9]. The majority of saltwater DGGE band sequences also fell into this cluster, including SW2-3, SW1-2, SW8-2 and SW6-2, sequences that represent a band shared across the majority of saltwater fingerprints (Fig. 3A). Cluster 3 was variable in composition, and contained both freshwater and saltwater DGGE band sequences. Reference *amoA* gene sequences in this cluster were from soil fosmid 54d9 [AJ627422; 13] and hotspring organisms *Candidatus* Nitrososphaera gargensis [ABY77595; 14] and *Candidatus* Nitrosocaldus yellowstonii [ABY83788; 15].

Cluster 4 was a distinct freshwater cluster; it was the largest of the clusters and contained the vast majority of the freshwater clone library and DGGE band sequences generated in this study. This cluster contained all sequences generated from a particular DGGE band that was shared over approximately half of the freshwater fingerprints (i.e. FW2-4, 6-11, 13, 14, 17, 19, 27; Fig. 3B). In addition, reference sequences from a variety of low-salinity environments fall into this cluster, including rivers, lakes, sediments, and wastewater, as well as the recently enriched *Candidatus* Nitrosoarchaeum limnia [16].

## Discussion

The present study has generated data that challenge decades of common knowledge regarding nitrogen cycling within aquarium filtration systems and solves outstanding questions remaining since *Nitrosomonas* spp. were undetected in freshwater aquaria [5]. From the sampling of 27 standalone freshwater aquaria from homes and retail outlets, our qPCR results have revealed a dominant AOA population in most of the freshwater filters sampled (Fig. 1). Indeed, 12 of the aquarium filters were associated only with an AOA *amoA* gene signal, despite using 40 cycles for the PCR. These results are important because they provide the first qualitative evidence that AOA may act alone in catalyzing ammonia oxidation. Though also generally dominated by AOA *amoA*, saltwater aquaria were more variable in the relative abundances of AOA and AOB, with both groups detected in each biofilter. Based on *amoA* gene abundances, the results of this study suggest that both AOA and AOB contribute to nitrification in saltwater aquaria and that AOA are the dominant contributors to nitrification in most established freshwater aquaria.

The results of this study also answer longstanding questions related to nitrification in aquarium biofilter environments. For example, the discovery that abundance of bacterial *amoA* genes may be orders of magnitude less than bacterial 16S rRNA genes (Table S1) provides an explanation for previous studies that were unable to detect *Nitrosomonas* spp. with gene probes [5], despite the ability to detect *Nitrosomonas* spp. with PCR primers for most samples [7]. Probe hybridization methods have limited detection for genes that represent less than 1% of the total community [17], whereas the high sensitivity of PCR can allow even a single copy of a gene to be detected if the amplification is not inhibited. Relative proportions of AOB in saltwater aquaria were generally greater than in freshwater biofilters (Fig. 1), which may explain the ability of both probe hybridization and PCR amplification to detect *Nitrosomonas* spp. in saltwater aquarium biofilters [5], [7], [8].

For a variety of genes in Bacteria, including both 16S rRNA and *amoA* genes, copy numbers within a cell are variable and often greater than one, and can therefore not be taken as a direct indication of population sizes. Conversely, available genomes from AOA representatives, including *Ca.* N. maritimus [18] and *Ca.* C. symbiosum [12], suggest that AOA cells contain one gene copy of each *amoA* and 16S rRNA. Whether all Thaumarchaeota possess ammonia monooxygenase genes is unknown. However, thaumarchaeal 16S rRNA genes have been detected that are up to 100 times more abundant than archaeal *amoA* genes [19], suggesting that some thaumarchaeal lineages do not gain energy by oxidizing ammonia. Some aquarium biofilters examined in this study (e.g. FW2, FW27, SW4; Table S1) yielded *amoA* gene copy numbers approximately equal to thaumarchaeal 16S rRNA copy numbers, implying that all Thaumarchaeota present in these aquaria possess *amoA* genes and presumably oxidize ammonia. Interestingly, other aquaria contained 16S rRNA genes that were orders of magnitude higher than AOA *amoA* genes (e.g. FW11, FW12, SW8; Table S1), which may imply to existence of thaumarchaeal lineages that do not oxidize ammonia.

AOA *amoA* gene diversity was variable across the filters collected in this study (Fig. 3A and B), with DGGE patterns ranging from relatively simple with few bands to complex with greater than ten discernible bands. In addition, many bands were shared across multiple aquarium filters from a variety of locations. Multidimensional scaling of DGGE fingerprints revealed that freshwater and saltwater fingerprints were largely separated in two-dimensional space. However, fingerprints of some freshwater samples were intermediate to major saltwater and freshwater clusters (Fig. 3C). DGGE band sequences derived from these intermediate fingerprints fell into a cluster of sequences containing approximately equal proportions of freshwater and saltwater clones (cluster 1, Fig. 4). Visual inspection of DGGE profiles (Fig. 3A and B) suggested a possible shift in *amoA* gene G+C content between freshwater and saltwater samples, and this was likely a factor in the separation observed. This trend was supported by clone libraries, which indicated that freshwater AOA *amoA* sequences had a higher average G+C content than their marine counterparts. The ecological implications of this finding are yet to be determined, but this poses an interesting hypothesis for future research. Multidimensional scaling of thaumarchaeal *amoA* clone library sequences (Fig. 4) revealed that freshwater and saltwater AOA *amoA* gene sequences largely cluster in distinct groups, which supports the separation observed in ordination analysis of DGGE profiles. Clusters were observed that predominantly contained saltwater sequences (i.e. clusters 2 and 3; Fig. 4), while one cluster (cluster 1; Fig. 4) contained approximately equal proportions of freshwater and saltwater sequences. Based on multidimensional scaling of both AOA *amoA* DGGE profiles and gene sequences, salinity appears to be a major factor in AOA differentiation. Nonetheless, these results suggest that at least some sequences are similar between environments, an observation that may indicate halotolerance of some phylotypes. Despite incomplete separation of saltwater and freshwater AOA *amoA* gene sequences, the majority (>80%) of freshwater sequences derived from clone libraries and DGGE bands clustered with sequences derived from a variety of freshwater environments, including lake sediments, wastewater, paddy soil, lakes and rivers (Fig. 4). This clustering confirms previous evidence for niche adaptation of aquatic freshwater AOA [e.g. 20], [21] and suggests that aquaria might provide valuable microcosms for investigating the ecology of aquatic AOA.

Although thaumarchaeal *amoA* gene copies may be several thousand fold more abundant than betaproteobacterial *amoA* genes in some marine [22] and terrestrial [23] environments, the relative contributions of AOA and AOB to environmental ammonia oxidation and the factors that affect their activity have been difficult to confirm. The concentration of ammonia may be a major factor affecting bacterial and thaumarchaeal ammonia oxidation. Recent studies have provided evidence that AOB are the dominant ammonia-oxidizing organisms in ammonia-rich soil and aquatic environments. For example, a recent study used stable-isotope labelled carbon dioxide and supplemented ammonia in soil to demonstrate that the labelled carbon was assimilated primarily into the nucleic acid of bacterial nitrifiers [24]. Foesel and colleagues (2008) found that *Nitrosomonas*-like AOB were numerically dominant in a marine aquaculture biofiltration system receiving high-ammonia influent (ranging from 340–1700 µg L^−1^). Further, the metabolic and numerical dominance of bacterial ammonia oxidizers in the presence of high ammonium concentrations is consistent with a previous study [7] investigating the eventual establishment of bacterial ammonia oxidizer colonization of ammonia-supplemented aquarium biofilters (5–60 mg NH_3_ L^−1^). In addition, most tested activated sludge bioreactors with high influent ammonia concentrations have been dominated by AOB populations [25], [26].

Predominance of AOA in low ammonia conditions has now been relatively well established in soil environments [27], [28], [29]. However, the role of ammonia in regulating ammonia-oxidizing populations in aquatic environments has not yet been well studied. That AOA are better adapted to low ammonia environments is supported by the present study with aquarium biofilters, where ammonium concentrations are maintained at consistently low concentrations, and certainly well below 1 mg N L^−1^ in all sampled aquaria (Table S1; Fig. 3). The majority of freshwater aquaria with very low ammonium concentrations (<100 µg L^−1^) were associated with increased proportions of AOA, and a significant inverse correlation was identified (Fig. 2 and Table S3). For statistical analyses, inclusion of additional samples with intermediate to high ammonia concentrations would have been preferable; however, established and maintained aquaria are typically kept at low ammonia concentrations to ensure fish health, and as a result we were unable to locate additional ammonia-rich aquaria within the timeframe of this study. The results of this study suggest that ammonia concentration in freshwater environments is an important parameter for determining the relative abundance of AOA and AOB. These results are consistent with other studies that have retrieved AOA *amoA* genes from low ammonia aquatic environments and demonstrated corresponding activity [30], [31], [32]. In addition, growth kinetics of *Ca.* N. maritimus str. SCM1 demonstrated a half saturation constant (*K*_m_) for ammonia that is substantially lower than for cultured AOB representatives [33] and similar to the measured ammonium concentrations associated with most of the aquaria sampled in this study (i.e. low µg L^−1^ ranges). However, because *Ca.* N. maritimus is the only AOA representative for which kinetic studies have been reported, it remains unclear whether all AOA are similarly adapted to oligotrophic conditions and demonstrate high substrate affinities for ammonia. For example, the recently isolated *Nitrososphaera viennensis* tolerates ammonium concentrations of up to 20 mM [34], which is considerably higher than the inhibitory concentrations of 2 to 3 mM that have been reported for *Ca.* N. maritimus and *Ca.* N. gargensis [14], [33].

Although the detectable ammonia concentrations in established freshwater aquaria are typically low as a result of biological ammonia oxidation, a preference for high ammonia concentrations by AOB suggests a possible role for their involvement in first establishing an aquarium when ammonia concentrations may approach levels associated with fish toxicity. In addition, ammonium concentration was positively and significantly correlated with the number of fish per gallon of aquarium water (Tables S2 and S3), suggesting that AOB may also be important for heavily stocked tanks that experience chronic high ammonia concentrations.

This study has identified that AOA are the dominant ammonia oxidizing microorganisms in freshwater aquarium biofilters. Aquarium ammonium concentrations were significantly and inversely correlated with AOA∶AOB ratios. Freshwater aquarium AOA *amoA* gene sequences largely clustered with other freshwater-associated sequences. This work provides a foundation for future studies of aquarium nitrification and AOA ecology. Aquaria may serve as valuable microcosms to investigate the factors affecting AOA and AOB dynamics in both natural and engineered aquatic communities, including wastewater treatment systems, aquaculture, lakes, rivers and oceans.

## Materials and Methods

### Sampling

Freshwater and saltwater aquarium biofilters were sampled from retail aquarium outlets and homes in Waterloo, Kitchener and Cambridge (Ontario, Canada) between June and December 2009 (Table S1). A total of 27 freshwater and 8 saltwater filters were analyzed in this study; filter types included sponge, floss, baffle, and live rock, and all filters collected were composed of cotton or synthetic polymeric material (e.g. nylon). Filter samples were collected using flamed forceps and scissors to cut small slices (e.g. 1 cm×1 cm×3 cm) from sponge material in external aquarium filtration systems. All filter samples were placed into 50-ml sterile tubes and stored on ice until returned to the laboratory within a few hours. Aquarium water samples were collected in 50-ml sterile tubes and stored on ice before being frozen at −80°C. Note that additional samples were collected for this study but were not included either due to poor yields of nucleic acid from the filter material or lack of amplification for both thaumarchaeal and bacterial *amoA* genes. A filter from one aquarium (FW27) was sampled four times over the course of two years to assess temporal stability in AOA/AOB ratios, and water was sampled several times (over six months and hourly over one day) to assess stability in ammonium concentrations within a given aquarium. The pH was assessed for all water samples with a DELTA 320 pH meter (Mettler Toledo, Columbus, OH). Ammonium concentrations were assessed fluorometrically, according to a previously published method [35] using a TD 700 fluorometer (Turner Designs, Sunnyvale, CA) and calculated from linear standard curves. Other water chemistry parameters were assessed with a simple water test kit available commercially (Quick Dip Aquarium Multi-Test Kit, Jungle Laboratories Corporation, Cibolo, TX). All sample information is contained within Table S1. We also sampled from eight saltwater aquarium filters and two aquarium supplements for comparison (Table S1). Bacterial supplements (typically bottled liquid suspensions) are intended to aid in populating newly established aquaria with active nitrifying bacteria, to help ensure that ammonia and nitrite concentrations remain below toxic levels during the initial 1–2 months of aquarium filter colonization. The aquarium supplements included were Cycle (SP1; Rolf C. Hagen Inc., Montreal, Canada), and Bio-Support (SP2; Big Al's Distribution Centre, Niagara Falls, NY).

### DNA extraction

A harsh nucleic acid extraction technique [36] was adapted to extract nucleic acids from thawed sponge filter material that had been cut into small fragments with flame-sterilized scissors. For supplements, aliquots (15 mL) of liquid aquarium supplements were pelleted by centrifugation at 7,000×g for 30 min, then suspended in lysis buffer for extraction. A beadbeating extraction was performed according to the published protocol with minor modifications. Nucleic acids were extracted from an equal volume of filter material rather than equivalent weight due to the variation in filter media porosity. The porous nature of filter material also required the phenol-chloroform-CTAB extraction buffer to be decanted away from the sponge material after cell lysis, prior to centrifugation and separation of aqueous and organic phases. To precipitate purified nucleic acids, two volumes of polyethylene glycol (PEG) solution (30% PEG 6000 and 1.6 M NaCl) were used in combination with linear polyacrylamide (AppliChem, Darmstadt, Germany) as a co-precipitant to avoid introducing exogenous DNA detected in commercial supplies of glycogen [37]. All extracts were separated on a 1% agarose gel with Gel Red nucleic acid stain (Biotium, Hayward, CA), visualized with an AlphaImager HP (Alpha Innotech Corporation, Santa Clara, CA) and quantified densitometrically by comparison to dilutions of known quantities of lamba DNA (New England Biolabs, Pickering, Canada) using AlphaView software (Alpha Innotech Corporation).

### Quantitative real-time PCR

Quantification of AOA and AOB *amoA* genes was performed using primers Arch-amoAF and Arch-amoAR [38] and amoA-1F and amoA-2R [39], respectively. Thaumarchaeal and bacterial 16S rRNA genes were quantified using primers 771F and 957R [40] and 341F and 518R [41], respectively. All real-time PCR amplifications were performed in duplicate with a reaction volume of 12.5 µl, which contained 2× iQ SYBR Green Supermix (Bio-Rad, Mississauga, Canada), 5 pmol of each primer, 5 µg of bovine serum albumin and 1 µl of template. Real-time PCR was performed on a CFX96 system (Bio-Rad). For both 16S rRNA genes, PCR conditions were 95°C for 3 min followed by 40 cycles of 95°C for 20 s, 55°C for 30 s and 72°C for 30 s (with fluorescence values recorded after the extension step). For *amoA* genes, the PCR conditions were the same as above, except with an extension time of 1 minute and annealing temperatures of 60°C and 58.5°C for bacterial and thaumarchaeal *amoA* genes, respectively. For all amplification reactions, melting curves from 65°C to 95°C were performed after each run with an incremental increase in temperature of 0.5°C.

PCR amplicons were used as standard template DNA, and were generated using the primers indicated above for their respective genes. We used genomic DNA from aquarium FW27 to generate standards for thaumarchaeal and bacterial *amoA* and thaumarchaeal 16S rRNA genes. Genomic DNA from *Escherichia coli* genomic DNA was used to generate bacterial 16S rRNA gene standards. Standard curves were constructed using serial dilutions of standard template DNA plotted against the cycle threshold (Ct) values for each dilution. Amplification efficiencies ranged from 90.6–98.2%, and coefficients of determinations (*R* ^2^) ranged from 0.988 to 0.999. Melting curves calculated for each target sequence showed single peaks and all PCR products were verified on a 1% agarose gel. Starting DNA copy numbers for each sample were calculated from the linear regression equation of each standard curve.

### Denaturing gradient gel electrophoresis

DGGE analysis of AOA *amoA* genes was performed as described previously [42] with minor modifications. Samples were run on 6% acrylamide gels, which provided better resolution than 8% acrylamide gels (data not shown). AOA *amoA* genes were amplified using primers CrenamoA23f and CrenamoA616r with thermal cycling as described elsewhere [43]. The DGGE system used was a DGGEK-2401 (C.B.S. Scientific Company, Del Mar, CA) using previously described technical modifications [11]. Gels were run for 15 h at 85 V and subsequently stained with SYBR green (Invitrogen) for 1 h. Gels were scanned using the Typhoon 9400 Variable Mode Imager (GE Healthcare, Piscataway, NJ). Individual DGGE bands were excised, amplified (using the above primers and conditions) and sequenced. From the original gel images, fingerprints were normalized for multi-gel alignment with GelCompar II (Applied Maths, Austin, TX) and a nonmetric multidimensional scaling (NMDS) plot was generated based on Pearson product moment correlations of background-subtracted densitometric curves.

### Clone libraries and ordination analysis

PCR amplicons for sequencing were generated using Arch-amoAF and Arch-amoAR, with thermal cycling as described previously [38]. Composites of freshwater and saltwater samples were produced by pooling equal nanogram amounts of PCR products from all freshwater and saltwater samples, respectively. Composite PCR products were ligated into the pGEM®-T Easy Vector (Promega, Madison, WI) according to the manufacturer's protocol. Single colonies were picked randomly and grown up in Luria Burtani broth containing ampicillin (100 µg ml^−1^), followed by a plasmid extraction and sequencing of inserts with the M13f primer. A total of 288 and 96 clones were sequenced for the composite freshwater and saltwater libraries, respectively.

All ordination analyses were performed using translated amino acid sequences. DNA sequences derived from both DGGE bands and clone libraries were translated using dna2pep [44]. After discarding sequences containing stop codons in the amino acid translation, a total of 261 freshwater clones and 84 saltwater clones remained. Reference sequences were obtained from GenBank for environmental clones as well as isolated or enriched AOA representatives. The collection of sequences was aligned using MUSCLE [45] and the resulting alignment was cropped so that all sequences spanned the same 160 amino acid region. All positions in the alignment were used for ordination analyses. A distance matrix was produced using protdist [46] and scaled by Kruskal's nonmetric multidimensional scaling using the MASS package [47]. All DNA sequences generated in this study were submitted to Genbank with accession numbers JN183456–JN183849.

### Statistical analyses

Pearson product-moment correlation coefficients and coefficients of determination were calculated in Excel 2010 (Microsoft, Redmond, WA), and associated p-values were calculated using InStat 3 (GraphPad Inc. San Diego, CA). Unpaired *t* tests were utilized to compare means in G+C content between freshwater and saltwater sequences and were conducted in InStat 3.

## Supporting Information

Figure S1 **Aquarium FW27 temporal patterns.** Ammonium concentrations (A) are shown over several months (during 2010), and hourly over a 12 hour period. Proportions of AOA/AOB in the FW27 sponge filter DNA extract are shown from four time points over the course of 2 years.
(PDF)

Table S1 Details of aquaria and associated quantitaive real-time PCR data.
(PDF)

Table S2 Pearson correlation coefficients for aquarium chemistry parameters and AOA/AOB abundances for all aquaria.
(PDF)

Table S3 Pearson correlation coefficients for aquarium chemistry parameters and AOA/AOB abundances for freshwater aquaria.
(PDF)
