# Supplementary material for: Aquarium Nitrification Revisited: Thaumarchaeota Are the Dominant Ammonia Oxidizers in Freshwater Aquarium Biofilters
Source: PLoS One. 2011 Aug 16;6(8):e23281. doi: 10.1371/journal.pone.0023281 (PMC3156731; doi:10.1371/journal.pone.0023281)
Supplement: Figure S1 — Aquarium FW27 temporal patterns. Ammonium concentrations (A) are shown over several months (during 2010), and hourly over a 12 hour period. Proportions of AOA/AOB in the FW27 sponge filter DNA extract are shown from four time points over the course of 2 years. (PDF) [file pone.0023281.s001.pdf]

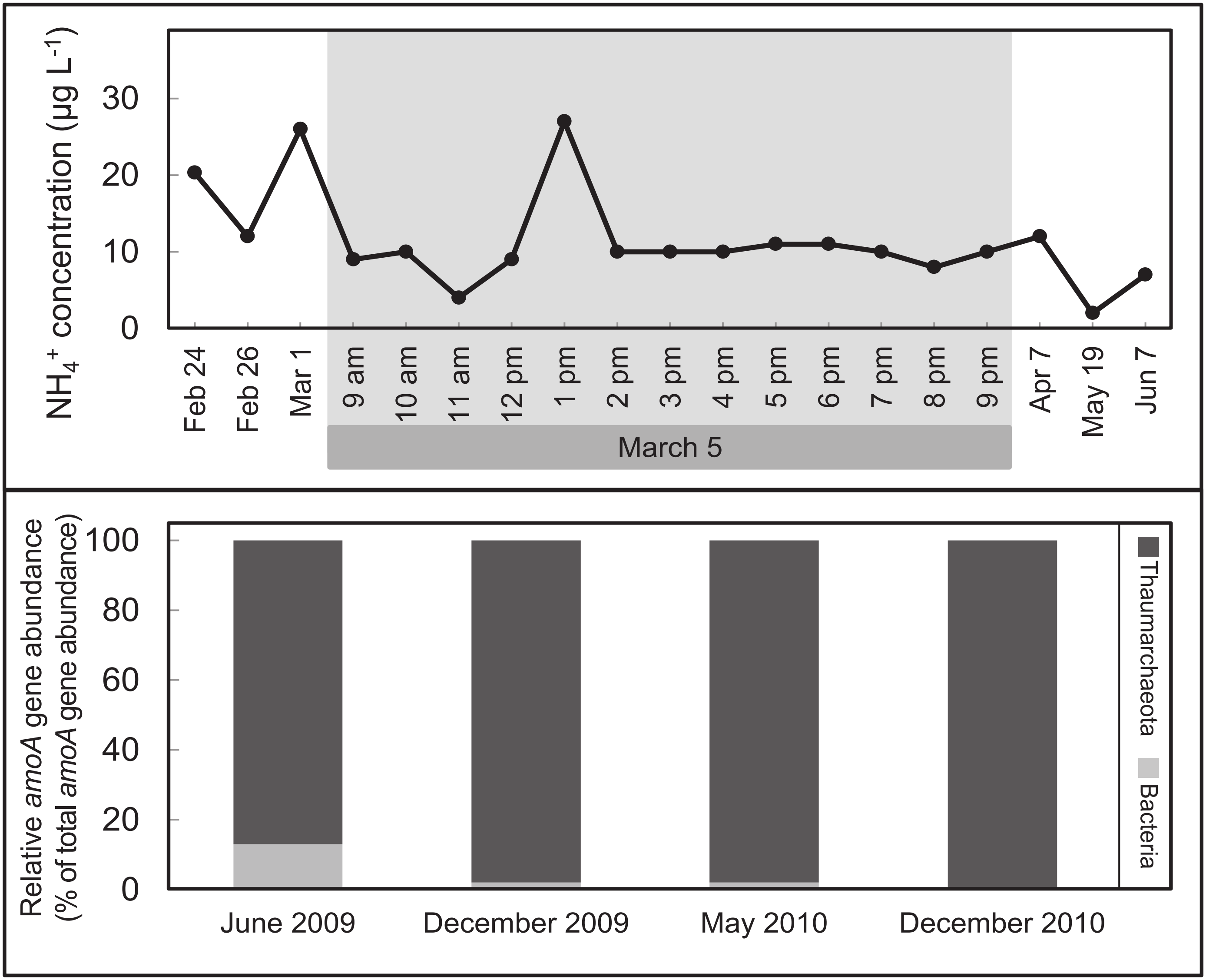

Figure S1. Aquarium FW27 temporal patterns. Ammonium concentrations (A) are shown over several months (during 2010), and hourly over a 12 hour period. Proportions of AOA/AOB in the FW27 sponge filter DNA extract are shown from four time points over the course of 2 years.
