# Supplementary material for: Aquarium Nitrification Revisited: Thaumarchaeota Are the Dominant Ammonia Oxidizers in Freshwater Aquarium Biofilters
Source: PLoS One. 2011 Aug 16;6(8):e23281. doi: 10.1371/journal.pone.0023281 (PMC3156731; doi:10.1371/journal.pone.0023281)
Supplement: Table S1 — Details of aquaria and associated quantitaive real-time PCR data. (PDF) [file pone.0023281.s002.pdf]

Table S1. Details of aquaria and associated quantitaive real-time PCR data.

			Thaumrchaeal *amoA*		Bacterial *amoA*		Thaumarchaeal 16S rRNA		Bacterial 16S rRNA															
Location	City	ID	Gene copies per ng gDNA	Std. Dev.	Gene copies per ng gDNA	Std. Dev.	Gene copies per ng gDNA	Std. Dev.	Gene copies per ng gDNA	Std. Dev.	Aquarium type	Size (gallons)	Approx. # of fish	NH_4+ (μg L^{-1})	pH	NO_3^- (ppm)	NO_2^- (ppm)	Hardness (ppm)	Alkalinity (ppm)	Live plants	Supplement used to start filter	Antobiotics in last 6 months	Filter type	Type of fish
Retail 1	Kitchener	SW1	710	645	1140	196	163265	3946	610398	381154	Salt	>100	15	133	8.24	20	0	NA	300	Yes	Unknown	No	Baffle	Mixed marine
Retail 1	Kitchener	SW2	2015	77	821	193	32002	3719	482665	65658	Salt/reef	>200	10	56	8.31	40	0	NA	40	Yes	Unknown	No	Live Rock	Reef (e.g. anemones, tangs)
Retail 1	Kitchener	SW3	212	27	63	5.4	3661	102	225042	25758	Salt/reef	>400	12	22	8.56	200	0	NA	300	Yes	Unknown	No	Baffle	Moray eel, shark
Retail 1	Kitchener	SW4	34	4	5.0	0.2	44	2.5	165868	5789	Salt	30	6	110	8.77	40	0	NA	120	No	Unknown	Yes	Sponge	Clownfish
Retail 2	Cambridge	SW5	434	92	155	37	292	5.8	369465	40869	Salt	110	25	77	8.58	20	0	NA	180	No	Unknown	Yes	Baffle	Mixed marine
Retail 2	Cambridge	SW6	69	23	82	8.0	7588	1204	399543	53000	Salt/reef	300	7	44	8.51	40	0	NA	300	Yes	Unknown	No	Baffle	Reef (e.g. anemones)
Retail 2	Cambridge	SW7	1606	117	87	13	4588	154	143187	10529	Salt	150	25	119	8.43	80	0	NA	300	Yes	Unknown	No	Baffle	Mixed marine
Retail 2	Cambridge	SW8	337	38	4223	26	164666	34482	350116	18458	Salt	110	5	36	8.39	160	0	NA	300	No	Unknown	No	Baffle	Mixed marine
Retail 1	Kitchener	FW1	346	5	3.7	0.8	117	4.7	256214	13124	Fresh	>250	>100	106	8.45	80	0.5	300	120	No	Unknown	No	Baffle	Mixed tropical
Retail 1	Kitchener	FW2	709	275	BDL	NA	758	168	177265	9905	Fresh	>250	>100	10	8.36	40	0	150	80	Yes	Unknown	No	Baffle	Mixed tropical
Retail 1	Kitchener	FW3	38	3	BDL	NA	197	12	95060	4490	Fresh/plant	>100	0	29	8.54	20	0	300	300	Yes	Unknown	No	Baffle	Plants only
Retail 1	Kitchener	FW4	3128	78	BDL	NA	5312	48	208012	5902	Fresh	110	25	24	8.58	200	0	300	120	No	Unknown	No	Sponge	African cichlids
Retail 2	Cambridge	FW5	BDL	NA	4.0	1.9	114	24	172519	53070	Fresh	110	20	445	7.85	200	2	300	40	No	Unknown	No	Sponge	Mixed tropical
Retail 3	Kitchener	FW6	119	20	50	8.5	1735	405	302936	80903	Fresh	15	10	164	8.63	20	0	300	300	No	Unknown	No	Floss	South American tropical
Retail 3	Kitchener	FW7	1562	62	BDL	NA	24577	733	214194	14074	Fresh	15	7	39	8.05	60	0	300	300	No	Unknown	No	Floss	Live bearers
Retail 3	Kitchener	FW8	209	33	4.7	0.2	549	12	159612	274	Fresh	15	5	0.9	8.00	20	0	300	180	No	Unknown	Yes	Sponge	Mixed tropical
Retail 3	Kitchener	FW9	148	0.4	5.4	0.6	392	38	159475	22177	Fresh	22	2	BDL	7.64	40	0	300	180	Yes	Unknown	No	Sponge	Mixed tropical
Retail 3	Kitchener	FW10	1242	114	599	3.3	11426	891	350549	32859	Fresh	22	>150	333	7.94	80	1	300	300	No	Unknown	No	Sponge	Goldfish
Retail 3	Kitchener	FW11	1800	577	5382	765	433105	42675	413670	13140	Fresh	65	300	559	7.71	40	2	300	300	No	Unknown	No	Sponge	Goldfish
Residential 1	Waterloo	FW12	42	0.5	926	18	10870	1066	296657	12395	Fresh	120	100	426	8.15	20	0	25	300	Yes	Yes	No	Sponge	African cichlids
Residential 1	Waterloo	FW13	376	28	BDL	NA	2281	10	111295	13940	Fresh	54	20	16	8.71	20	0	300	300	Yes	Yes	No	Sponge	African cichlids
Residential 2	Kitchener	FW14	389	78	BDL	NA	6726	650	257006	41257	Fresh	110	10	41	8.74	20	0	300	120	No	No	No	Sponge	South American tropical
Residential 2	Kitchener	FW15	150	24	BDL	NA	1693	54	172204	7170	Fresh	40	8	30	8.38	160	0.5	150	300	Yes	Yes	No	Sponge	South American cichlids
Residential 2	Kitchener	FW16	230	26	BDL	NA	9321	426	755417	59916	Fresh	30	4	69	7.95	200	3	150	80	Yes	Yes	No	Sponge	Killifish
Residential 2	Kitchener	FW17	1261	69	BDL	NA	7342	697	876849	121988	Fresh	100	12	66	7.91	40	0	150	0	No	No	No	Sponge	Cichlids, Loach, Catfish
Residential 2	Kitchener	FW18	531	25	BDL	NA	11802	426	172741	2817	Fresh	12	5	20	9.21	60	0	75	300	Yes	No	No	Sponge	Corydoras, Killifish
Residential 3	Kitchener	FW19	191	24	4.3	0.2	3416	239	52770	7630	Fresh	30	14	7	8.11	120	0	300	80	Yes	Yes	No	Sponge	Mixed tropical
Residential 3	Kitchener	FW20	23	2	38	0.4	70	11	165467	3139	Fresh	10	3	17	8.65	40	0	300	180	No	No	No	Sponge	Mixed tropical
Residential 3	Kitchener	FW21	86	13	5.4	0.1	1644	47	147223	7798	Fresh	15	15	45	8.34	200	2	300	100	No	No	No	Sponge	Mixed tropical
Residential 4	Kitchener	FW22	395	17	4.5	1.5	7371	334	172139	15213	Fresh	30	2	44	7.93	10	0	150	40	Yes	No	No	Sponge	South American cichlids
Residential 4	Kitchener	FW23	32	14	3.7	0.9	5882	151	241338	134289	Fresh	75	40	139	8.02	40	0	300	120	Yes	No	No	Sponge	South American cichlids
Residential 4	Kitchener	FW24	2482	270	6.5	0.4	5078	48	250438	23138	Fresh	25	25	12	8.20	40	0	150	0	Yes	No	No	Sponge	African cichlids
Residential 5	Waterloo	FW25	2160	179	BDL	NA	3944	55	112034	3240	Fresh	10	4	20	7.93	10	0	300	180	Yes	Yes	No	Sponge	Mixed tropical
Residential 5	Waterloo	FW26	1138	143	12	4.2	10161	236	210381	2803	Fresh	5	3	11	7.80	10	0	300	120	Yes	No	No	Sponge	Mixed tropical
Residential 6	Waterloo	FW27	30180	3156	BDL	NA	37536	5094	253781	15544	Fresh	110	15	20	8.39	60	0	225	120	No	No	No	Sponge	African cichlids
Bio-Support	NA	SP1	BDL	NA	13586	443	BDL	NA	945223	4331	NA	NA	NA	NA	NA	NA	NA	NA	NA	NA	NA	NA	NA	NA
Cycle	NA	SP2	BDL	NA	43950	17070	BDL	NA	137850	8718	NA	NA	NA	NA	NA	NA	NA	NA	NA	NA	NA	NA	NA	NA

BDL: below detection limit

NA: not applicable

gDNA: genomic DNA
