# Supplementary material for: Aquarium Nitrification Revisited: Thaumarchaeota Are the Dominant Ammonia Oxidizers in Freshwater Aquarium Biofilters
Source: PLoS One. 2011 Aug 16;6(8):e23281. doi: 10.1371/journal.pone.0023281 (PMC3156731; doi:10.1371/journal.pone.0023281)
Supplement: Table S2 — Pearson correlation coefficients for aquarium chemistry parameters and AOA/AOB abundances for all aquaria. (PDF) [file pone.0023281.s003.pdf]

Table S2. Pearson correlation coefficients for aquarium chemistry parameters and AOA/AOB abundances for all aquaria.

	% AOA *amoA*	% AOB *amoA*	NH_4^+	NO_3^-	NO_2^-	pH	alkalinity	Hardness	fish $gallon^{-1}$
**% AOA *amoA***	--								
**% AOB *amoA***	-1.00	--							
**NH_4^+**	-0.67	0.67	--						
**NO_3^-**	-0.07	0.07	0.01	--					
**NO_2^-**	-0.21	0.21	0.48	0.52	--				
**pH**	-0.31	0.31	-0.34	0.13	-0.35	--			
**alkalinity**	-0.30	0.30	0.20	-0.03	-0.14	0.28	--		
**hardness**	NA	NA	NA	NA	NA	NA	NA	--	
**fish $gallon^{-1}$**	-0.21	0.21	0.60	-0.04	0.36	-0.31	0.24	NA	--

Legend: white = No significance; light grey = $p < 0.05$; dark grey = $p < 0.001$
(Shaded: -0.67 and 0.67 in the NH_4^+ row, and 0.60 in the fish $gallon^{-1}$ row, are dark grey; 0.48 and 0.52 in the NO_2^- row are light grey.)
