# Supplementary material for: Aquarium Nitrification Revisited: Thaumarchaeota Are the Dominant Ammonia Oxidizers in Freshwater Aquarium Biofilters
Source: PLoS One. 2011 Aug 16;6(8):e23281. doi: 10.1371/journal.pone.0023281 (PMC3156731; doi:10.1371/journal.pone.0023281)
Supplement: Table S3 — Pearson correlation coefficients for aquarium chemistry parameters and AOA/AOB abundances for freshwater aquaria. (PDF) [file pone.0023281.s004.pdf]

Table S3. Pearson correlation coefficients for aquarium chemistry parameters and AOA/AOB abundances for freshwater aquaria.

	% AOA *amoA*	% AOB *amoA*	NH_4^+	NO_3^-	NO_2^-	pH	alkalinity	hardness	fish $gallon^{-1}$
**% AOA *amoA***	--								
**% AOB *amoA***	-1.00	--							
**NH_4^+**	-0.85	0.85	--						
**NO_3^-**	-0.04	0.04	0.06	--					
**NO_2^-**	-0.04	0.04	0.48	0.61	--				
**pH**	0.20	-0.20	-0.34	0.14	-0.31	--			
**alkalinity**	-0.20	0.20	0.26	-0.16	-0.10	0.28	--		
**hardness**	0.05	-0.05	-0.03	-0.09	0.04	-0.14	0.07	--	
**fish $gallon^{-1}$**	-0.33	0.33	0.61	-0.04	0.33	-0.27	0.35	0.16	--

No significance

$p < 0.05$

$p < 0.001$
